# High Dietary Fructose Intake on Cardiovascular Disease Related Parameters in Growing Rats

**DOI:** 10.3390/nu9010011

**Published:** 2016-12-26

**Authors:** SooYeon Yoo, Hyejin Ahn, Yoo Kyoung Park

**Affiliations:** 1 Department of Medical Nutrition, Kyung Hee University, 1732 Deogyeong-daero, Giheung-gu, Yongin 17104, Korea; sooyeon928@hanmail.net (S.Y.Y.); hjahn@khu.ac.kr (H.A.); 2 Research Institute of Medical Nutrition, Kyung Hee University, 26, Kyungheedae-ro, Dongdaemun-gu, Seoul 02447, Korea

**Keywords:** high fructose diet, cardiovascular disease (CVD), growing rat

## Abstract

The objective of this study was to determine the effects of a high-fructose diet on cardiovascular disease (CVD)-related parameters in growing rats. Three-week-old female Sprague Dawley rats were randomly assigned to four experimental groups; a regular diet group (RD: fed regular diet based on AIN-93G, *n* = 8), a high-fructose diet group (30Frc: fed regular diet with 30% fructose, *n* = 8), a high-fat diet group (45Fat: fed regular diet with 45 kcal% fat, *n* = 8) or a high fructose with high-fat diet group (30Frc + 45Fat, fed diet 30% fructose with 45 kcal% fat, *n* = 8). After an eight-week treatment period, the body weight, total-fat weight, serum glucose, insulin, lipid profiles and pro-inflammatory cytokines, abdominal aortic wall thickness, and expressions of eNOS and ET-1 mRNA were analyzed. The result showed that total-fat weight was higher in the 30Frc, 45Fat, and 30Frc + 45Fat groups compared to the RD group (*p* < 0.05). Serum triglyceride (TG) levels were highest in the 30Frc group than the other groups (*p* < 0.05). The abdominal aorta of 30Frc, 45Fat, and 30Frc + 45Fat groups had higher wall thickness than the RD group (*p* < 0.05). Abdominal aortic eNOS mRNA level was decreased in 30Frc, 45Fat, and 30Frc + 45Fat groups compared to the RD group (*p* < 0.05), and also 45Fat and 30Frc + 45Fat groups had decreased mRNA expression of eNOS compared to the 30Frc group (*p* < 0.05). ET-1 mRNA level was higher in 30Frc, 45Fat, and 30Frc + 45Fat groups than the RD group (*p* < 0.05). Both high fructose consumption and high fat consumption in growing rats had similar negative effects on CVD-related parameters.

## 1. Introduction

Sugar-sweetened beverages and processed foods are the main source of fructose [1]. According to The Korea National Health and Nutrition Examination Survey (KNHNES) [2], consumption of beverage products increased 3.7 folds from 1998 (45.5 ± 2.0 g) to 2014 (167.4 ± 5.2 g). Based on data collected from a 2010 study from The Korea Health Industry Development Institute (KHIDI) [3], consumption of sugar-sweetened beverages and processed foods in adolescents were higher than in the adult group. 

Recently, a considerable volume of research was performed in both animals and humans dedicated to clarify the link between dietary fructose and health risk markers such as obesity and cardiovascular disease. Consumption of sugar-sweetened beverages has a positive correlation with body weight gain [4,5] in human, and in animals [6,7,8]. Bocarsly et al. [9] reported that rats fed with water containing high fructose corn syrup for eight weeks had increased body weight and body fat, and Crescenzo et al. [10] showed significant increases in body fat in rats that consumed fructose for eight weeks, with no significant difference in body weight. These observations are particularly important because accumulation of body fats leads to an increase of pro-inflammatory cytokines (TNF-α, IL-6, PAI-1) [11].

In addition, a high fructose diet is known to lead hypertension and insulin resistance in animals [12,13]. Insulin resistance has been proposed as an underlying mechanism that links endothelial dysfunction factors such as endothelial nitric oxide synthase (eNOS) and Endothelin-1 (ET-1) [14]. Also, chronic exposure of dyslipidemia has a major effect on cardiovascular disease (CVD) [15]. De Castro et al. [16] reported that rats fed with a high fructose diet had significantly increased levels of in serum total-cholesterol and triglyceride. These changes are significantly associated with an increased incidence of cardiovascular disease [17].

Changes of CVD-related parameters in childhood is correlated with development into CVD which affects CVD risks later in life [18]. Although high-fructose affects CVD-related parameters in adult human and animals, these effects have not been investigated in adolescent or growing animals. Therefore, this study investigates the effects of a high-fructose diet and compares the results with a high-fat diet on CVD-related parameters in growing rats.

## 2. Materials and Methods

### 2.1. Experimental Design and Diet

The experimental protocol was approved by the Animal Care Use Review Committee of Kyung Hee University (IACUC, protocol number: KHP-2014-01-1). Three-week-old female Sprague-Dawley rats (*n* = 32) were provided by SLC, Inc. (Shizuoka, Japan). Rats were housed individually in polycarbonate cages in temperature-controlled rooms (22 ± 2 °C) with a relative humidity of 55% ± 5%, and a 12-h light/dark cycle. The rats were fed a pellet chow diet, and given water ad libitum for an adaptation period of 10 days. All rats were weighed weekly, and food intake was measured daily. After a 10-day adaption period, animals were randomized selected into the four different groups: regular diet group (RD) (*n* = 8) rats were fed an AIN-93G (D10012G, Research Diets Inc., New Brunswick, NJ, USA) diet, high fructose diet group (30Frc) (*n* = 8) rats were fed a 30% fructose (D14010101, Research Diets Inc.) diet, high fat diet group (45Fat) (*n* = 8) rats were fed a 45 kcal% fat as soy bean oil and lard (D14010102, Research Diets Inc.) diet, and high fat diet with high fructose diet group (30Frc + 45Fat) (*n* = 8) rats were fed a diet with 30% fructose and 45 kcal% fat (D14010103, Research Diets Inc.). All animals were maintained on these diets ad libitum for eight weeks. Composition of experimental diets is shown in Table 1.

### 2.2. Body Weight, Food Consumption, and Fat Mass

Body weights and food consumption were measured weekly and daily, respectively. The food efficiency ratio (FER) was calculated using the following formula: (weight gain (g)/week)/(food consumed (kcal)/week). At the end of the eight-week experimental period, total fat was removed and weighed immediately after killing. The total fat was measured by combining the weight of subcutaneous fat and visceral fat.

### 2.3. Analysis of Blood Parameters

Blood was collected at the end of experiment, following a 12-h overnight fast. Rats were anesthetized with a small amount of ethyl ether, and blood samples were taken by heart puncture. Blood samples were immediately collected into serum-separating tubes (SST) and were centrifuged at 3000 rpm for 15 min at 4 °C. Serum was stored at −70 °C until used in assays. Serum triglyceride (TG), Total cholesterol (T-Chol), HDL-cholesterol (HDL-C), and glucose levels were determined using commercial kits (Asan Co. Ltd., Seoul, Korea). Atherogenic Index (AI) was calculated using the following formula: AI = (total cholesterol − HDL-cholesterol)/HDL-cholesterol. Serum Insulin concentrations were determined using an ELISA rat/mouse insulin kit (ALPCO Diagnostics, Salem, NH, USA). Insulin resistance was calculated by a homeostasis assessment model (HOMA-IR) and calculated from fasting insulin and glucose concentration according to the formula: (fasting insulin (ng/mL) × fasting glucose (mg/dL))/22.5. Quantitative insulin sensitivity check index (QUICKI) was also calculated using the following formula: QUICKI = 1/(log fasting insulin (mg/dL) + log fasting glucose(mg/dL)). Pro-inflammatory cytokines (TNF-α, IL-6, and PAI-1) were measured in duplicate using Milipore’s MILLIPLEX rat CVD cytokine panel (Millipore, Billerica, MA, USA). 

### 2.4. Analysis of Abdominal Aorta

Abdominal aorta samples were obtained after rats were sacrificed at the end of experiment. The abdominal aortas were fixed in a 4% formalin solution, followed by sequential dehydration (70% ethanol, 100% ethanol, and acetone), xylene clearance, and paraffin embedding. The paraffin-embedded aortas were cut into 5-micron slices with a microtome (820 II; Reichert-Jung, Bensheim, Germany). Then the sliced aortas were stained with Harris hematoxylin and eosinY (H & E). Wall thickness and lumen diameter of abdominal aortas were determined on five isotropic uniform random sections per animal at a magnification of 500:1 and phase contrast. All sections were photographed using a microscope (model Axiovert S100, Zeiss, Oberkochen, Germany) connected to a camera (model AxioCam, Zeiss) and MetaMorph software (Molecular Devices, Sunnyvale, CA, USA). Wall thickness and lumen diameter were determined as the mean of the minimal and maximal value. 

### 2.5. Quantitative Real-Time Polymerase Chain Reaction (PCR)

Total RNA was extracted from abdominal aorta using an RNeasy mini kit (Qiagen, Gaithersburg, MD, USA) according to the manufacturer’s instructions. Real-time quantitative PCR was performed using an Applied Bio-systems (Applied Bio-systems, Foster City, CA, USA); 1 μL with 100 nM of each primer (forward and reverse) reaction consisted of 10 μL of the SYBR Green Super-mix (iQ SYBR Green Super-mix, Bio-Rad Laboratories Inc. Hercules, CA, USA), according to the manufacturer’s instructions. Thermal cycling was initiated by denaturation at 95 °C for 10 min, followed by 40 cycles of 95 °C for 15 s and 60 °C for 30 s, then an annealing step was performed at adequate temperature in function of the primers and 72 °C for 30 s for extension. After the final cycle, melting curves were monitored from 55 to 65 °C (0.05 °C/s). The primer sequences were: rat eNOS, 5′-CAACAAACCGAGGCAATCTTC-3′ (forward), 5′-CCCGGCCAGCGTAGCT-3′ (reverse), and rat ET-1, 5′-TGGACATCATCTGGGTCAACA-3′ (forward), 5′-GCTTAGACCTAGAAGGGCTTCCTAGT-3′ (reverse), and rat glyceraldehyde 3′-phosphate dehydrogenase (GAPDH), 5′-TGGCCTCCAAGGAGTAAGAAAC-3′ (forward), 5′-GGCCTCTCTCTTGCTCTCAGTATC-3′ (reverse). The detected genes were eNOS and ET-1. 

### 2.6. Statistical Analysis

All measurements were performed in duplicate, and statistical calculations were performed with Statistical Package for the social Sciences (SPSS, version 20.0, IBM Corp., Armonk, NY, USA) software. All data were presented as mean ± SD. Differences in measured parameters among the experimental groups were analyzed by the one-way ANOVA and Duncan’s multiple range tests. The respective effects of operation and diet were analyzed by two-way ANOVA. The differences were considered to be significant when the *p* value was less than 0.05.

## 3. Results

### 3.1. Body Weights, Food Intakes, Calorie Intakes, and Food Efficiency Ratios

The effect of the diet on body weight, body weight gain, food intake, calorie intake, and food efficiency ratio is shown in Table 2. 

The initial body weights were not significantly different among the experimental groups. Feeding of high fat diet groups (45Fat, 30Frc + 45Fat) resulted in a significantly higher body weight than the RD and 45Fat groups (278.1 ± 22.1 g, 277.2 ± 16.6 g vs. 251.3 ± 9.6 g, 263.3 ± 15.0 g, *p* < 0.05). The food intake was significantly higher in the RD (12.5 ± 0.8 g/day) and 30Frc (13.2 ± 1.0 g/day) groups compared to that of 45Fat (11.3 ± 0.9 g/day) and 30Frc + 45Fat (11.5 ± 0.8 g/day) groups (*p* < 0.05). However, total calorie intakes and food efficiency ratios were not significantly different among the experimental groups. Total calorie was calculated from food intake (g) × calorie density (kcal/g). 

### 3.2. Total-Fat Weights

**Figure 1:**
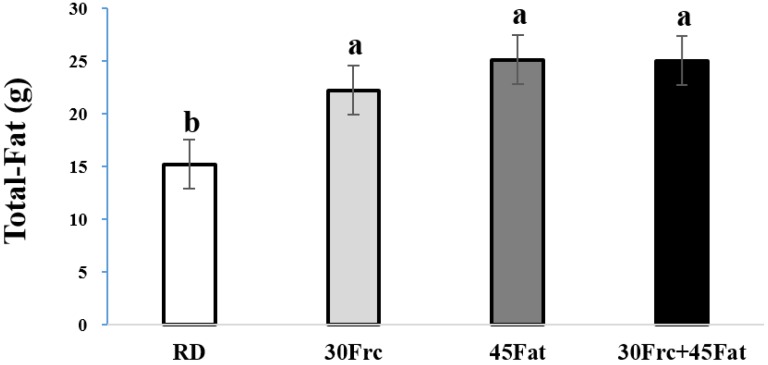
Total-fat weight in the experimental groups. RD: rats received a regular diet based on AIN-93G (4.0 kcal/g diet); 30Frc: rats received a 30% fructose-diet based on control-diet (4.0 kcal/g diet); 45Fat: rats a received 45 kcal% fat-diet (4.8 kcal/g diet); 30Frc + 45Fat: rats received a 45 kcal% fat-diet with 30% fructose (4.8 kcal/g diet). Statistical differences between the experimental groups were based on one-way ANOVA and Duncan’s multiple range tests at *p* < 0.05. Means with different alphabetical letters are significantly different (*p* < 0.05).

The total-fat weights of the experimental groups are shown in Figure 1.

The average total-fat weights were 15.2 ± 3.6, 22.2 ± 3.8, 25.1 ± 3.5, and 25.0 ± 2.4 g in the RD, 30Frc, 45Fat, and 30Frc + 45Fat groups. The total-fat weights were significantly higher in 30Frc, 45Fat, and 30Frc + 45Fat groups than the RD group (*p* < 0.05). 

### 3.3. Serum Levels of Glucose and Insulin, HOMA-IR, and QUICKI

The blood glucose and insulin levels, HOMA-IR, and QUICKI are shown in Table 3. The serum glucose and insulin levels did not differ among the experimental groups. Also, no differences were observed in HOMA-IR and QUICKI among groups.

### 3.4. Serum Levels of Lipid Profiles

The serum lipid profile levels are shown in Table 4. Serum Total-C, HDL-C, and atherogenic index were not different among groups. However, the serum TG levels in the 30Frc group (108.6 ± 25.2 mg/dL) were significantly higher than the RD, 45Fat, and 30Frc + 45Fat groups (71.4 ± 19.8, 88.6 ± 26.9, and 93.9 ± 24.1 mg/dL, respectively) (*p* < 0.05).

### 3.5. Serum Levels of Pro-Inflammatory Cytokines 

The serum pro-inflammatory cytokines levels are shown in Table 5. The mean levels of serum pro-inflammatory cytokines levels (TNF-α, IL-6, PAI-1) did not differ among the experimental groups.

### 3.6. Analysis of Abdominal Aorta Wall Thickness and Lumen Diameter 

**Figure 2:**
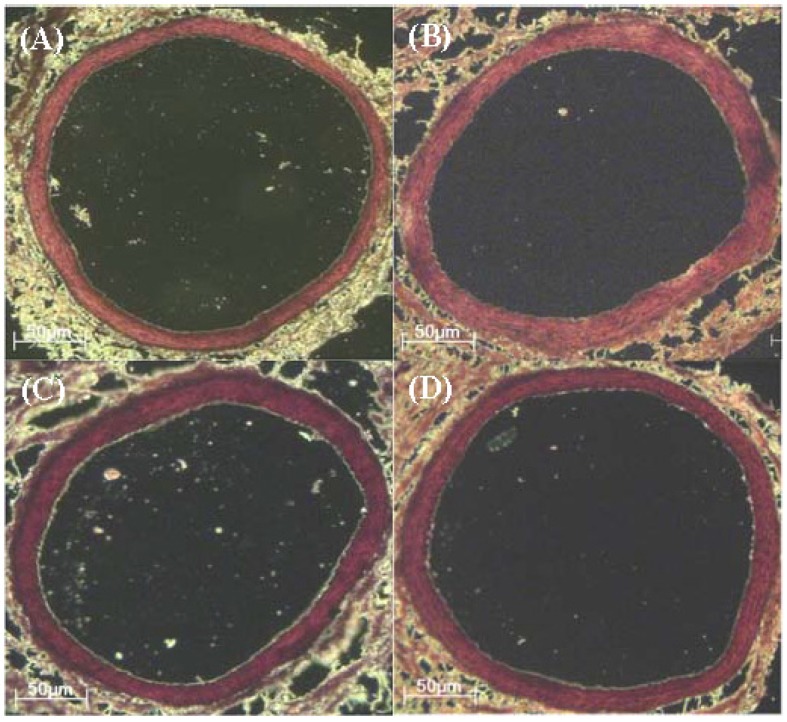
Abdominal aortic wall thickness and lumen diameter taken at eight weeks, pertaining to the respective groups (**A**) RD; (**B**) 30Frc; (**C**) 45Fat; (**D**) 30Frc + 45Fat. Hematoxylin and eosin (H & E) stained abdominal aorta × 500. Magnification bars 50 μm. RD: rats received a regular diet based on AIN-93G (4.0 kcal/g diet); 30Frc: rats received a 30% fructose-diet based on control-diet (4.0 kcal/g diet); 45Fat: rats received a 45 kcal% fat-diet (4.8 kcal/g diet); 30Frc + 45Fat: rats received a 45 kcal% fat-diet with 30% fructose (4.8 kcal/g diet).

The abdominal aorta wall thickness and lumen diameter are shown in Table 6 and Figure 2. The aorta wall thickness was significantly increased in 30Frc, 45Fat, and 30Frc + 45Fat groups (23.6 ± 0.9 μm, 23.7 ± 2.8 μm, and 22.5 ± 2.2 μm, respectively, *p* < 0.05) than the RD group (18.5 ± 0.5 μm). The lumen diameter was not different among the groups. The wall thickness:lumen ratio was increased in 30Frc, 45Fat and 30Frc + 45Fat groups (0.096 ± 0.001 μm, 0.098 ± 0.014 μm, and 0.085 ± 0.005 μm, respectively, *p* < 0.05) than the RD group (0.061 ± 0.009 μm).

### 3.7. Abdominal Aortic eNOS and ET-1 mRNA Expression Measured by qRTPC

**Figure 3:**
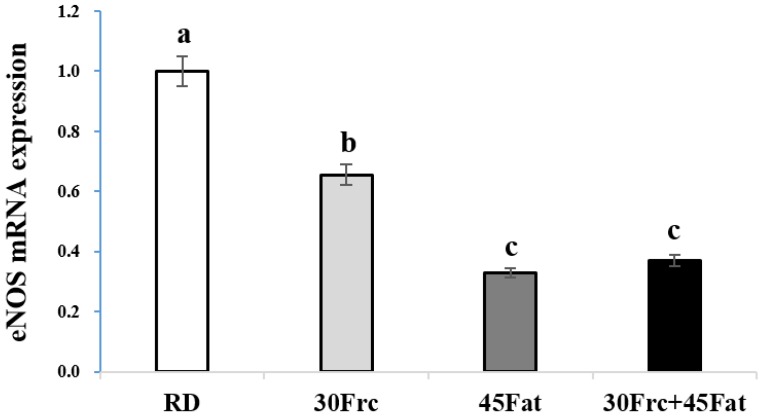
Aortic eNOS mRNA expression measured by quantitative real-time polymerase chain reaction (qRTPCR). RD: rats received a regular diet based on AIN-93G (4.0 kcal/g diet); 30Frc: rats received a 30% fructose-diet based on control-diet (4.0 kcal/g diet); 45Fat: rats received a 45 kcal% fat-diet (4.8 kcal/g diet); 30Frc + 45Fat: rats received a 45 kcal% fat-diet with 30% fructose (4.8 kcal/g diet). Statistical differences between the experimental groups were based on one-way ANOVA and Duncan’s multiple range tests at *p* < 0.05. Means with different alphabetical letters are significantly different (*p* < 0.05).

**Figure 4:**
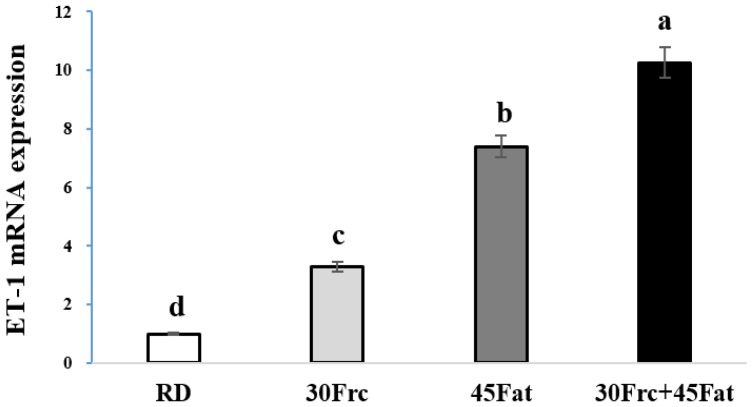
Aortic ET-1 mRNA expression measured by qRTPCR. RD: rats received a regular diet based on AIN-93G (4.0 kcal/g diet); 30Frc: rats received a 30% fructose-diet based on control-diet (4.0 kcal/g diet); 45Fat: rats received a 45 kcal% fat-diet (4.8 kcal/g diet); 30Frc + 45Fat: rats received a 45 kcal% fat-diet with 30% fructose (4.8 kcal/g diet). Statistical differences between the experimental groups were based on one-way ANOVA and Duncan’s multiple range tests at *p* < 0.05. Means with different alphabetical letters are significantly different (*p* < 0.05).

The abdominal aortic eNOS and ET-1 mRNA expression of the experimental groups are shown in Figure 3 and Figure 4. The aortic eNOS mRNA expression was significantly decreased in 45Fat and 30Frc + 45Fat groups (0.3 ± 0.06 and 0.4 ± 0.07, respectively, *p* < 0.05) compared to the RD and 30Frc groups (1.0 ± 0.00 and 0.7 ± 0.05, respectively). Also, for the 30Frc group that was fed the high fructose diet constantly, the aortic eNOS mRNA expression was significantly decreased compared to the RD group (*p* < 0.05). The aortic ET-1 mRNA expression of the 30Frc + 45Fat group was the highest among the four groups (10.3 ± 1.8 vs. 1.0 ± 0.4, 3.3 ± 0.3, and 7.4 ± 0.7, respectively, for the RD, 30Frc, and 45Fat groups, *p* < 0.05). 

## 4. Discussion

The purpose of this study was to determine the effects of a high-fructose diet on cardiovascular disease (CVD)-related parameters in growing rats. 

It was known that rats fed high-fat diets increased body weight and body fat [19,20]. The present study showed that the consumption of a high-fat diet (45Fat, 30Frc + 45Fat) in growing rats significantly increased body weight and body fat compared to regular diet and high-fructose diet groups. The high-fructose diet did not affect the body weight, whereas it significantly increased the weight of body fat compared to the regular diet fed group. The previous study reported that rats fed with the high-fructose diet (60%) for 10 weeks increased body weight [7]. However, Crescenzo et al. [10] showed increased white adipose tissue (WAT) in rats fed fructose (60%), despite no difference in body weight gain. An increase in body weight alone does not necessarily indicate obesity, it has to be considered along with other factors, such as changes in body composition [21]. The increase in body fat reflected the obesogenic property. This suggests that obesity can be induced by high-fructose diet, as well as high-fat diet. 

Dyslipidemia, especially hypertriglyceridemia and insulin resistance are major factors associated with CVD in rats fed a high-fructose diet (60%) [22,23]. Our data showed that the 30Frc group had significantly increased serum TG levels compared to the other groups. In the liver, fructose is divided into glyceraldehyde and dihydroxyacetone phosphate, ultimately becoming triglyceride [24]. Therefore, exposure to high fructose levels to rapidly increased levels of triglyceride synthesis. We consider that excessive fructose consumption can lead to dyslipidemia and obesity; these changes are caused CVD. 

Insulin resistance is closely linked to dyslipimenia [25]. Many previous studies have reported that the high-fructose diet can induce hyperglycemia (35%, 66%) [26,27]. However, this study showed no difference in serum glucose and insulin levels. The previous study suggested that although fructose does not appear to acutely increase insulin levels, chronic exposure seems to indirectly cause insulin resistance and obesity through other mechanisms, such as GLUT5 fructose transporters and inflammation [28]. Iida et al. [29] reported that rats fed 40% fructose for eight weeks displayed no difference in serum insulin levels. Another study showed that rats fed with 66% fructose for two weeks increased plasma TG, even though there was no change in plasma glucose, insulin, and body weight [30]. Increased triglyceride levels in response to high-fructose diet could have resulted in insulin resistance by reducing the insulin signaling pathway [27]. Our data, together with the previously established literature showed, suggest that chronic exposure to a high-fructose diet can result in hypertriglycemia, and that this change could cause insulin resistance. 

Enlarged body fat induces the expression of pro-inflammatory cytokines [11]. A previous study reported that a high fructose diet can increase hepatic mRNA expression of TNF-α, IL-6, and the weight of epididymal fat pads in rats [31]. However, the present study had shown that rats fed a high-fructose diet significantly increased the total body fat, but the serum pro-inflammatory cytokines (TNF-α, IL-6, PAI-1) did not change in between groups. Chronic inflammation is an important pathogenic factor in the development of CVD [32,33]. Large amounts of markers for inflammatory cytokines can be released from the adipose tissue [34]. Yudkin et al. [35] reported that adipose tissue can also synthesize pro-inflammatory cytokines such as TNF-α and IL-6. In this way, increased body fat itself promotes inflammation. According to this hypothesis, high fructose consumption leads to increased body fat and obesity, which can cause changes to inflammatory cytokines. In the present study we showed that rats fed a high-fructose diet significantly increased their total-fat weight, although the serum pro-inflammatory cytokines did not change. 

Changes in the aorta wall thickness is significantly associated with serum lipid profiles and hypertension, which begins in childhood and may develop into cardiovascular disease [35,36,37,38]. According to a previous study, the tunica intima-media layer was increased in the high-fructose diet group [31]. Autopsy studies have reported that the first atherosclerotic lesions actually begin to develop in the abdominal aorta [39]. Therefore, we chose the abdominal aorta to conduct the present experiment. In the present study we showed that the abdominal aorta of high-fructose diet and high fat diet rats were thicker in comparison to the regular diet group. We consider that high-fructose consumption as well as high-fat consumption, can have a significant effect on abdominal aorta wall thickness in the growing rats. These early changes can possibly lead to endothelial dysfunction.

Endothelial dysfunction is a systemic disorder in the pathogenesis of atherosclerosis [40], which plays an important role in hypertension [41] and CVD [42]. The endothelium maintains the balance between vasoconstriction and vasodilation, but when this balance is disrupted, endothelial dysfunction occurs which can be lead to CVD [43]. A major vasodilator NO is synthesized by nitric oxide synthases [44]. Endothelial dysfunction may occur as a result of decreased eNOS activity or reduced bioavailability of NO [14]. The endothelium also produces vasoconstrictors, such as endothelin and angiotension II. Endothelin is the most potent endogenous vasoconstrictor [43]. As such, in this study, we analyzed vasodilator eNOS and vasoconstrictor ET-1. A previous study showed that NO synthesis inhibited rats had elevated blood pressure [45]. Endemann et al. [14] reported that the eNOS activity of rats fed high-fructose diet decreased in the aorta. In the present study, mRNA expression of eNOS in the abdominal aorta in 45Fat and 30Frc + 45Fat groups was significantly decreased in comparison to those of the other groups. Additionally, the 30Frc group had significantly decreased mRNA expression of eNOS compared to the RD group. ET-1 is an important vasoconstrictor produced by endothelial cells that contributes to enhanced blood pressure [43,46]. A previous study reported that rats fed a high-fructose diet for nine weeks had increased ET-1 levels [47]. Similarly, our study showed that the abdominal aortic ET-1 mRNA expression in 30Frc, 45Fat, and 30Frc + 45Fat groups were significantly higher than the RD group. Our data, together with the previously established literature, suggest that high-fructose diet, as well as the high-fat diet, negatively affected endothelium-derived relaxing and contracting factors. These changes are important factors for the development of hypertension and vascular dysfunction.

## 5. Conclusions

Collectively, the high-fructose diets increased the total-fat weight and serum TG levels in growing rats. Additionally, it had negative effects on abdominal aortic thickness and eNOS, ET-1 mRNA expression. 

The strength of our study is that we fed 30% fructose-diets to the animals. Numerous animal studies have used extreme doses of ~60% fructose, which, as White [48] suggested, show results that are not physiological, and likely cause abnormal metabolism, and therefore cannot be depended on to assess human risk. In the present study we were able to show that 30% fructose can induce total-fat weight gain, increase aorta wall thickness, and affect eNOS and ET-1 mRNA expression, which are related to CVD risk factors. One potential limitation in the present study was that the sample size was small, which may lessen the significance of the results. 

In conclusion, we confirmed high fructose consumption, as well as high fat consumption, in growing rats had negative effects on CVD-related parameters, such as total-fat weight, serum TG, aorta wall thickness, and eNOS, ET-1 mRNA expression of abdominal aorta.

## Figures and Tables

**Table 1:** Ingredient composition of experimental diets.

	RD		30Frc		45Fat		30Frc + 45Fat	
**%**	**g**	**kcal**	**g**	**kcal**	**g**	**kcal**	**g**	**kcal**
Protein	20	20	20	20	24	20	24	20
Carbohydrate	64	64	64	64	41	35	41	35
Fat	7	16	7	16	24	45	24	45
Total		100		100		100		100
kcal/gm	4.0		4.0		4.8		4.8	
**Ingredient**	**g**	**kcal**	**g**	**kcal**	**g**	**kcal**	**g**	**kcal**
Casein, 80 Mesh	200	800	200	800	200	800	200	800
l-Cystine	3	12	3	12	3	12	3	12
Corn Starch	397.5	1590	229.5	918	137	548	0	0
Maltodextrin 10	132	528	100	400	100	400	37	148
Sucrose	100	400	0	0	100	400	0	0
Fructose	0	0	300	1200	0	0	300	1200
Cellulose	50	0	50	0	50	0	50	0
Soybean Oil	70	630	70	630	26	234	26	234
*t*-Butylhydroquinone	0.014	0	0.014	0	0.014	0	0.014	0
Lard	0	0	0	0	174	1566	174	1566
Mineral Mix	35	0	35	0	35	0	35	0
Vitamin Mix	10	40	10	40	10	40	10	40
Choline Bitartrate	2.5	0	2.5	0	2.5	0	2.5	0
Total	1000	4000	1000	4000	837.5	4000	837.5	4000

RD: rats received a regular diet based on AIN-93G (4.0 kcal/g diet); 30Frc: rats received a 30% fructose-diet based on regular diet (4.0 kcal/g diet); 45Fat: rats received a 45 kcal% fat-diet (4.8 kcal/g diet); 30Frc + 45Fat: rats received a 45 kcal% fat -diet with 30% fructose (4.8 kcal/g diet).

**Table 2:** Body weights, food intakes, calorie intakes, and food efficiency ratio.

	RD (*n* = 8)	30Frc (*n* = 8)	45Fat (*n* = 8)	30Frc + 45Fat (*n* = 8)
Initial body weight (g)	74.6 ± 2.6	74.9 ± 5.0	74.5 ± 4.71	74.4 ± 4.1
Final body weight (g)	251.3 ± 9.6 ^b^	263.3 ± 15.0 ^a,b^	278.1 ± 22.1 ^a^	277.2 ± 16.6 ^a^
Weight gain (g)	177.2 ± 9.8 ^b^	188.0 ± 13.4 ^a,b^	204.3 ± 22.6 ^a^	201.9 ± 17.7 ^a^
Food intake (g/day)	12.5 ± 0.8 ^a^	13.2 ± 0.9 ^a^	11.3 ± 0.9 ^b^	11.5 ± 0.8 ^b^
Calorie intake (kcal/day)	49.8 ± 2.9	52.4 ± 4.4	53.8 ± 4.6	54.7 ± 4.7
Food efficiency ratio	0.05 ± 0.00	0.05 ± 0.00	0.05 ± 0.01	0.05 ± 0.00

All values are presented as means ± standard deviation (SD). RD: rats received a regular diet based on AIN-93G (4.0 kcal/g diet); 30Frc: rats received a 30% fructose-diet based on control-diet (4.0 kcal/g diet); 45Fat: rats received a 45 kcal% fat-diet (4.8 kcal/g diet); 30Frc + 45Fat: rats received a 45 kcal% fat-diet with 30% fructose (4.8 kcal/g diet); Food efficiency ratio = (weight gain (g)/week)/(food consumed (kcal)/week). Statistical differences between the experimental groups were based on one-way ANOVA and Duncan’s multiple range tests at *p* < 0.05. Means with different alphabetical superscripts are significantly different (*p* < 0.05).

**Table 3:** Serum levels of glucose and insulin.

	RD (*n* = 8)	30Frc (*n* = 8)	45Fat (*n* = 8)	30Frc + 45Fat (*n* = 8)
Glucose (mg/dL)	143.6 ± 10.3	142.0 ± 9.2	141.4 ± 14.9	136.6 ± 15.8
Insulin (ng/mL)	1.2 ± 1.4	2.2 ± 1.7	0.9± 0.3	1.3 ± 0.9
HOMA-IR	8.1 ± 9.1	12.7 ± 11.7	5.9± 2.4	8.3 ± 6.2
QUICKI	0.5 ± 0.07	0.4 ± 0.07	0.5± 0.05	0.5 ± 0.07

All values are presented as means ± standard deviation (SD). RD: rats received a regular diet based on AIN-93G (4.0 kcal/g diet); 30Frc: rats received a 30% fructose-diet based on control-diet (4.0 kcal/g diet); 45Fat: rats received a 45 kcal% fat-diet (4.8 kcal/g diet); 30Frc + 45Fat: rats received a 45 kcal% fat-diet with 30% fructose (4.8 kcal/g diet), HOMA-IR: homoeostasis model assessment of insulin resistance was calculated by (fasting insulin (ng/mL); QUICKI: 1/(log(fasting insulin ng/mL) + log(fasting glucose mg/dL)) × fasting glucose (mg/dL))/22.5. There were no significant differences among groups according to ANOVA and Duncan’s multiple range tests.

**Table 4:** Serum levels of lipid profiles.

	RD (*n* = 8)	30Frc (*n* = 8)	45Fat (*n* = 8)	30Frc + 45Fat (*n* = 8)
TG (mg/dL)	71.4 ± 19.8 ^b^	108.6 ± 25.2 ^a^	88.6 ± 26.9 ^a,b^	93.9 ± 24.1 ^a,b^
Total-C (mg/dL)	107.9 ± 19.7	114.6 ± 17.5	97.8 ± 15.4	96.3 ± 18.4
HDL-C (mg/dL)	98.1 ± 12.4	103.8 ± 10.9	92.1 ± 13.4	91.8 ± 14.7
AI	0.09 ± 0.07	0.10 ± 0.05	0.06 ± 0.03	0.05 ± 0.05

All values are presented as means ± standard deviation (SD). RD: rats received a regular diet based on AIN-93G (4.0 kcal/g diet); 30Frc: rats received a 30% fructose-diet based on control-diet (4.0 kcal/g diet); 45Fat: rats received a 45 kcal% fat-diet (4.8 kcal/g diet); 30Frc + 45Fat: rats received a 45 kcal% fat-diet with 30% fructose (4.8 kcal/g diet); TG: Triglyceride; Total-C: Total cholesterol; AI: Atherogenic index = (total cholesterol − HDL-cholesterol)/HDL-cholesterol. Statistical difference between the experimental groups were based on one-way ANOVA and Duncan’s multiple range tests at *p* < 0.05. Means with different alphabetical superscripts are significantly different (*p* < 0.05).

**Table 5:** Serum levels of pro-inflammatory cytokines.

	RD (*n* = 8)	30Frc (*n* = 8)	45Fat (*n* = 8)	30Frc + 45Fat (*n* = 8)
TNF-α (pg/mL)	55.1 ± 4.9	51.5 ± 5.5	47.0 ± 4.8	48.3 ± 10.9
IL-6 (pg/mL)	11.6 ± 4.2	18.2 ± 11.6	12.6 ± 4.0	15.5 ± 8.3
PAI-1 (pg/mL)	100.8 ± 13.3	101.5 ± 9.4	96.0 ± 4.8	99.4 ± 20.5

All values are presented as means ± standard deviation (SD). RD: rats received a regular diet based on AIN-93G (4.0 kcal/g diet); 30Frc: rats received a 30% fructose-diet based on control-diet (4.0 kcal/g diet); 45Fat: rats received a 45 kcal% fat-diet (4.8 kcal/g diet); 30Frc + 45Fat: rats received a 45 kcal% fat-diet with 30% fructose (4.8 kcal/g diet); TNF-α: Tumor Necrosis α; IL-6: Interleukin-6; PAI-1: Plasminogen activator inhibitor-1. There were no significant differences among groups according to ANOVA and Duncan’s multiple range tests.

**Table 6:** Abdominal aorta wall thickness and lumen diameter.

	RD (*n* = 8)	30Frc (*n* = 8)	45Fat (*n* = 8)	30Frc + 45Fat (*n* = 8)
Wall thickness (μm)	18.5 ± 0.5 ^b^	23.6 ± 0.9 ^a^	23.7 ± 2.8 ^a^	22.5 ± 2.2 ^a^
Lumen diameter (μm)	306.8 ± 54.4	248.4 ± 25.1	243.9 ± 29.4	266.9 ± 29.0
Wall thickness/ Lumen ratio (μm/μm)	0.061 ± 0.009 ^b^	0.096 ± 0.001 ^a^	0.098 ± 0.014 ^a^	0.085 ± 0.005 ^a^

All values are presented as means ± standard deviation (SD). RD: rats received a regular diet based on AIN-93G (4.0 kcal/g diet); 30Frc: rats received a 30% fructose-diet based on control-diet (4.0 kcal/g diet); 45Fat: rats received a 45 kcal% fat-diet (4.8 kcal/g diet); 30Frc + 45Fat: rats received a 45 kcal% fat-diet with 30% fructose (4.8 kcal/g diet). Statistical differences between the experimental groups were based on one-way ANOVA and Duncan’s multiple range tests at *p* < 0.05. Means with different alphabetical superscripts are significantly different (*p* < 0.05).
